# Human Epididymis Secretory Protein 4 (HE4) Compromises Cytotoxic Mononuclear Cells via Inducing Dual Specificity Phosphatase 6

**DOI:** 10.3389/fphar.2019.00216

**Published:** 2019-03-19

**Authors:** Nicole E. James, Matthew T. Oliver, Jennifer R. Ribeiro, Evelyn Cantillo, Rachael B. Rowswell-Turner, Kyu-Kwang Kim, Clinton O. Chichester, Paul A. DiSilvestro, Richard G. Moore, Rakesh K. Singh, Naohiro Yano, Ting C. Zhao

**Affiliations:** ^1^Program in Women's Oncology, Department of Obstetrics and Gynecology, Women & Infants Hospital, Warren Alpert Medical School of Brown University, Providence, RI, United States; ^2^Department of Pharmacy, University of Rhode Island, Kingston, RI, United States; ^3^Division of Gynecologic Oncology, Department of Obstetrics and Gynecology, Wilmot Cancer Institute, University of Rochester Medical Center, Rochester, NY, United States; ^4^Department of Surgery, Roger Williams Medical Center, Boston University Medical School, Providence, RI, United States

**Keywords:** HE4, ovarian cancer, DUSP6, CD8 T cells, tumor immunology

## Abstract

While selective overexpression of serum clinical biomarker Human epididymis secretory protein 4 (HE4) is indicative of ovarian cancer tumorigenesis, much is still known about the mechanistic role of the HE4 gene or gene product. Here, we examine the role of the secretory glycoprotein HE4 in ovarian cancer immune evasion. Through modified subtractive hybridization analyses of human peripheral blood mononuclear cells (PBMCs), we have characterized gene targets of HE4 and established a preliminary mechanism of HE4-mediated immune failure in ovarian tumors. Dual specificity phosphatase 6 (DUSP6) emerged as the most upregulated gene in PBMCs upon *in vitro* exposure to HE4. DUSP6 was found to be upregulated in CD8^+^ cells and CD56^+^ cells. HE4 exposure reduced Erk1/2 phosphorylation specifically in these cell populations and the effect was erased by co-incubation with a DUSP6 inhibitor, (E)-2-benzylidene-3-(cyclohexylamino)-2,3-dihydro-1H-inden-1-one (BCI). In co-culture with PBMCs, HE4-silenced SKOV3 human ovarian cancer cells exhibited enhanced proliferation upon exposure to external HE4, while this effect was partially attenuated by adding BCI to the culture. Additionally, the reversal effects of BCI were erased in the co-culture with CD8^+^ / CD56^+^ cell deprived PBMCs. Taken together, these findings show that HE4 enhances tumorigenesis of ovarian cancer by compromising cytotoxic CD8^+^ and CD56^+^ cells through upregulation of self-produced DUSP6.

## Introduction

Epithelial ovarian cancer (EOC) is the fifth leading cause of cancer death in women, and the deadliest of all gynecologic cancers. The American Cancer Society estimates that in 2017, there will be an estimated 22,440 new cases of EOC and 14,080 deaths in the United States^1^ Unfortunately, only 15% of patients are diagnosed at an early stage when the disease is fundamentally curable, keeping the 5-year survival rate at a dismal 46%^2^. Recurrence following initial treatment is common, occurring in approximately 80% of cases, with all recurrent disease patients eventually succumbing to their illness (Kim et al., 2012). These dire statistics highlight the need for continued research into improved diagnostic and treatment options for EOC patients.

Despite continued efforts, there remains a lack of effective treatments for EOC. Standard first-line therapy consists of debulking surgery followed by taxane-platinum chemotherapy (Kim et al., 2012). While targeted therapies such as bevacizumab and olaparib are approved to treat EOC, these treatments have not led to an improvement in overall survival (Yap et al., 2009). One promising new area of investigation lies in understanding how tumors develop immune tolerance and evade elimination by cytotoxic lymphocytes. Immune checkpoint molecules such as PD-1, CTLA4, TIM3, IDO, and others act to suppress T cell activation, therefore helping tumor cells escape immune targeting and elimination (Zhao and Subramanian, 2018). Nivolumab, a monoclonal antibody that binds to PD-1, preventing it from binding to its tumor cell associated ligands, PDL1/PDL2, has greatly improved survival for metastatic melanoma patients (Volpe et al., 2017). Anti- PD-1 therapies have also been studied in relapsed platinum-resistant EOC; however, overall response rates do not exceed 15% (Mittica et al., 2016). The inefficacy of immune checkpoint inhibitors observed in EOC is likely due to compensatory immune suppressive pathways (Curran et al., 2010; Holmgaard et al., 2013) or activation of oncogenic pathways that promote immune tolerance (Zhao and Subramanian, 2018). Overall, a greater understanding of factors that contribute to immune evasion in EOC is required in order to develop treatments that have the ability to reactivate the body's immune response to tumors.

Human epididymis protein-4 (HE4) is a member of the whey acidic four-disulfide core protein family (Bingle et al., 2002). HE4 is elevated in tumor tissue and serum of EOC patients, and is included in the Risk of Ovarian Malignancy Algorithm (ROMA) score, along with the biomarker CA125 and menopausal status. The ROMA score is used in the diagnosis and management of EOC (Hellström et al., 2003; Moore et al., 2009). ROMA shows greater sensitivity and specificity for the detection and monitoring of EOC than the Risk of Malignancy Index, which uses CA125, pelvic sonography, and menopausal status (Moore et al., 2009). HE4 also has the advantage of presenting fewer false positives than CA125 in the case of benign gynecologic disorders (Hellström et al., 2003; Moore et al., 2012). *In vitro* and *in vivo* studies have shown that HE4 promotes multiple aspects of ovarian cancer aggression, including tumor growth, proliferation, metastasis, chemoresistance, and anti-estrogen resistance (Lu et al., 2012; Zhuang et al., 2013, 2014; Zhu et al., 2013, 2016; Lokich et al., 2014; Moore et al., 2014; Wang et al., 2015; Ribeiro et al., 2016; Lee et al., 2017). Clinically, patients with high levels of serum HE4 are more chemoresistant to traditional platinum-based therapies and exhibit a poorer prognosis (Angioli et al., 2014; Chudecka-Głaz et al., 2014; Moore et al., 2014; Vallius et al., 2014). Our group has also hypothesized that HE4 may play a role in the promotion of immune evasion in EOC. We determined that HE4 has the ability to mediate gene expression in peripheral blood mononuclear cells (PBMCs), and then evaluated HE4's effect on one of its identified targets, DUSP6, ultimately investigating how this relationship affects immune cytotoxicity against ovarian cancer cells.

## Materials and Methods

### Subtractive Hybridization and TA-cloning

5 × 10^7^ PBMCs from single donor were suspended in 5 mL of serum free RPMI1640 medium (Invitrogen, 31800) and incubated with or without 0.01 μg/mL of rHE4 (Abcam, ab184603) for 6 h, and total RNA was isolated using TRIzol™ Reagent (Invitrogen, 15596018). Next, mRNA was purified using Magnosphere™ UltraPure mRNA Purification Kit (Takara-Clontech, 9186). From the 5 μg of mRNA, subtractive cDNA libraries were constructed using PCR-Select™ cDNA Subtraction Kit (Takara-Clontech, 637401) following the manufacturer's protocols (Figure S1A). PCR products of the differentially expressed genes were cloned into a pUC19-TA vector. Top 10 competent cells (Invitrogen, C404003) were transformed with the clones and were seeded on Xgal/IPTG containing LB/ampicillin plates. The colonies of clones containing the inserts were selected by blue/white selection and were amplified by direct colony PCR using LA Taq® DNA polymerase (Takara-Clontech, RR002A) and M13 primers (Table S1). PCR products in the range of 200 to 3000 bp were then subjected to direct sequencing (Figures S1B,C).

### Cell Culture

Primary human PBMCs were obtained under the auspices of Women & Infants Hospital IRB approval from total blood of four individual volunteers by density gradient centrifugation using Histopaque®-1077 (Sigma-Aldrich, 10771). The human ovarian tumor cell line, SKOV3, human NK cell line, NK-92MI, and human T cell lines, TALL-104 and H9, were obtained from ATCC (HTB-77, CRL-2408, CRL-11386 and HTB-176, respectively). RPMI1640 was used for culturing PBMCs and lymphocyte lines. DMEM (Invitrogen, 31600) was used to culture SKOV3 cells. Conditioned media was obtained from 24-h PBMC culture. Residual rHE4 in the conditioned media was deprived as follows: 5 mL of media was incubated with 10 μg (100 μL) of anti-human HE4 antibody (Santa Cruz Biotechnology, sc-293473) for 1 h at 4°C. Then, 100 mL packed volume of protein G coated sepharose beads (GE Healthcare Life Science, 17061801) were added to the media and incubated for 4 h at 4°C. After the incubation, the sepharose beads were removed by centrifugation and the supernatants were processed through a sterile 0.2 μm pore syringe filter. Concentrations of HE4 in the conditioned media were confirmed by ELISA (Table S2). For the cell-mediated cytotoxicity assay, 1 × 10^6^ /well (6-well plates for caspase-3 western blotting), 5 × 10^5^/well (4-chamber slide for Ki-67 immunostaining) or 1 × 10^3^/well of (96-well plates for proliferation assay) target cells (SKOV3) were seeded and incubated overnight with complete media. The next day, cells were placed in serum free media for another overnight incubation and then effector cells (PBMCs) were added. The ratio of the effector cells to the target cells was decided based on a previously published study (Song et al., 2012). In that study, various ratios of PBMC:SKOV3 (80:1, 40:1, 20:1 or 10:1) were applied for the cell mediated cytotoxicity assay. In the present study, considering an *in vivo* environment where there would be more tumor cells than the infiltrating mononuclear cells, we chose a lower PBMC ratio (5:1). Some of the cultures contained 0.01 μg/mL of rHE4 and 1 μM of the DUSP6 inhibitor (E)-2-benzylidene-3-(cyclohexylamino)-2,3-dihydro-1H-inden-1-one (BCI; Sigma-Aldrich, B4313). After a12-h incubation, the effector cells were washed away and SKOV3 cells were evaluated for proliferation indicated by either Ki67 staining or cleaved caspase-3 levels. All experiments were performed under serum free condition.

### Flow Cytometry

FITC-labeled anti CD3, CD4, CD8, CD14, CD19, and CD56 antibodies were obtained from BD Biosciences (555916, 561005, 560960, 555397, 555412, and 562794, respectively). Alexa Fluor® 647-conjugated anti DUSP6 antibody was obtained from Abcam (ab200751). Alexa Fluor®647-conjugated anti phosphor-p44/42 MAPK antibody was obtained from Cell Signaling Technology (13148). After staining for cell surface markers (CD3, CD14, CD19 and CD56) the cell membrane was permeabilized by 0.2% Triton X-100 and 0.2% digitonin, and then stained for DUSP6 or phosphor-p44/42-MAPK. FITC-labeled mouse IgG1κ (BD55748) and Alexa Fluor® 647-labeled mouse IgG1κ (BD55783) were used as isotype controls. Flow cytometric analysis was performed with FACSCanto system and FACSDiva software (BD Biosciences).

### Quantitative Real-Time PCR

RNA was isolated from cells using TRIzol (Invitrogen, A33250) according to the manufacturer's instructions. cDNA was synthesized using SuperScript III reverse transcriptase (Invitrogen, 18080093). qPCR was performed using Premix Ex-Taq™ II (Clontech-Takara, 639676) probes for DUSP6. All reactions were normalized using GAPDH as an endogenous control. Amplification data were analyzed using the ΔΔCt method. Sequences of PCR primers are summarized in Table S1.

### ELISA

ELISA kits for HE4 and DUSP6 were obtained from My BioSource (MBS280223 and MBS073193, respectively). Assays were performed following the manufacturer's instruction.

### Western Blotting

Phosphorylation of Erk1/2 in NK-92MI, TALL-104 and H9 cell lines were assessed by western blotting. Apoptosis in SKOV3 cells was evaluated as the ratio of cleaved caspase-3 to intact caspase-3 on the western blotting gel. Antibodies against phosphorylated total Erk1/2 MAPK and cleaved caspase-3 were obtained from Cell Signaling Technology (9101 4695 and 9661). An antibody against intact caspase-3 was obtained from Abcam (ab13847). The results were visualized with SuperSignal™ West Pico chemiluminescent substrate (Thermo Fisher Scientifics, 34080) and analyzed with the UN-SCAN-IT gel software for Windows (Silk Scientific Inc.).

### HE4 Silencing With shRNA

shRNA for human HE4 (Origene, TR318721) were transfected into SKOV3 using Lipofectamine® 2000 (Invitrogen, 11668) following the manufacture's instruction. Individual single cells were selected by culturing under the pressure of 5 μg/mL of puromycin (Research Products International, 58-58-2). Phenotypes of the clones were evaluated by western blotting using anti-HE4 antibody (Origene, TA801137; Figure S2).

### Cell Proliferation Assay

1 × 10^3^/well of SKOV3 cells were seeded in each well of a 96-well culture plate. After overnight incubation with serum free medium, 5 × 10^3^/well of effector cells (PBMCs) were added to the quiescent cells. After 12-h incubation, the effector cells were washed away and cells were incubated under serum free conditions for 24, 48, and 72 h. Cell proliferations were evaluated using fluorescent-based CEllTiter-Blue® (Promega, G8080) and Spectra Max Gemini EM fluorescent micro plate reader (Molecular Devices).

### Immunohistochemistry

5 × 10^5^/chamber of SKOV3 cells were seeded in a 4-chamber slide. After overnight incubation with serum free medium, 2.5 × 10^6^/chamber of effector cells (PBMCs) were added to the quiescent cells and the cells were cultured for 48 h. Ki67 positive cells were counted in 20 of 200x fields. Affinity purified normal mouse IgG1 (Santa Cruz Biotechnology, sc-3877) was used as a negative control. A mouse anti-Ki67 monoclonal antibody was purchased from BD Biosciences (550609). An alkaline phosphatase (ALP) labeled anti-mouse IgG secondary antibody and an ALP substrate kit were obtained from Vector laboratories (AP-2000, SK-5100).

### Depletion of CD8^+^ and CD56^+^ Cells From PBMCs

CD8^+^ and CD56^+^ cells were removed from PBMCs using magnetic CD8 and CD56 MicroBeads (Miltenyi Biotec, 130-045-201 and 130-050-401) with autoMACS cell separator (Miltenyi Biotec, 130-092-545). Briefly, 5 × 10^7^ of PBMCs were suspended in 60 μL of separation buffer (PBS, pH 7.2 with 0.5% BSA and 2 mM EDTA), and then 20 μL each of CD8 and CD56 MicroBeads were added, followed by a 15 min incubation at 4°C. After washing, cells were resuspended in 500 μL of the separation buffer and magnetic separation was performed using autoMACS® Columns (Miltenyi Biotec, 130-021-101). Unlabeled cells that passed through were collected and combined with total effluent from the washed column. Efficacy of the depletion was evaluated by two-color flow cytometry using FITC-labeled anti-CD8 antibody (BD Biosciences, 560960) and PE-labeled anti-CD56 antibody (BD Biosciences, 561903, Figure S3).

### Statistics

Data was expressed as an average ± SEM of at least four independent experiments. An unpaired, two-tailed Student *t*-test was used to determine significance. Multiple treatments were analyzed by using one-way ANOVA followed by Ryan's multiple comparison test. Differences between groups were considered statistically significant when *p* < 0.05.

## Results

### Differential Expression of PBMC Genes After HE4 Exposure

To identify differentially expressed genes after HE4 exposure, modified subtractive hybridization was performed as depicted in Figure S1A. PCR products of the differentially expressed genes were cloned into pUC19-TA vectors to create a differential cDNA library (Figures S1B,C). PCR products from 250 HE4-induced and HE4-suppressed gene colonies were sequenced, resulting in the identification of 209 induced genes and 206 suppressed genes. Among the identified genes, 20 induced and 13 suppressed sequences showed no significant similarity (NSS) to known genes in available nucleotide databases. Among the 209 induced genes, dual specificity phosphatase 6 (DUSP6) emerged as one of the most frequently identified genes (3 times out of 250 sequences, 1.2%; Table 1).

**Table 1:** Genes induced in response to HE4.

**Frequency**	**ID**	**Gene name**
20	NSS	No significant similarity
3	NG_033915	Dual specificity phosphatase 6 (DUSP6)
3	XM_017002424	Capping actin protein of muscle Z line alpha sub unit 1 (CAPZA1)
3	NM_001402	Eukaryotic translation elongation factor 1 alpha 1 (EEF1A1)
3	XM_017000674	FGR proto-oncogene, SRC family tyrosine kinase (FGR)
3	NM_001261446.1	Thioredoxin reductase 1 (TXNRD1)
3	NM_021109	Thymosin beta 4, X-linked (TMSB4X)
3	BC006364	Tubulin folding cofactor D
2	AK223032	Beta actin variant
2	AC008397.7	Chromosome 19 clone CTC-251H24
2	NM_001170330	Chromosome 4 open reading frame 3 (C4orf3)
2	AY430097	DAZ associated protein 2 (DAZAP2)
2	NM_001005360	Dynamin 2 (DNM2)
2	NG_002350.4	Eukaryotic translation elongation factor 1 alpha 1 pseudogene 5 (EEF1A1P5)
2	NM_004468.4	Four and a half LIM domains 3 (FHL3)
2	NM_001077488	GNAS complex locus (GNAS)
2	NM_001321232	Histocompatibility (minor) HA-1 (HMHA1)
2	NM_000206.2	Interleukin 2 receptor, gamma (IL2RG)
2	NM_001127605.2	Lipase A, lysosomal acid (LIPA)
2	NM_012335.3	Myosin IF (MYO1F)
2	XM_011541520	Notch 2 (NOTCH2)
2	NM_001165412	Nuclear factor of kappa light polypeptide gene enhancer in B-cells 1 (NFKB1)
2	NM_020820.3	Phosphatidylinositol-3,4,5-trisphosphate dependent Rac exchange factor 1 (PREX1)
2	NM_001251855	Phosphoinositide-3-kinase regulatory subunit 5 (PIK3R5)
2	NM_201384.2	Plectin (PLEC)
2	NM_002952	Ribosomal protein S2 (RPS2)
2	NM_001007.4	Ribosomal protein S4, X-linked (RPS4X)
2	NM_000655	Selectin L (SELL)
2	NM_004252	SLC9A3 regulator 1 (SLC9A3R1)
2	NM_022733.2	Small ArfGAP2 (SMAP2)
2	NM_001278206	Solute carrier family 43, member 3 (SLC43A3)
2	NM_025250.2	Tweety family member 3 (TTYH3)
2	BC050652.1	Zinc finger, DHHC-type containing 16
2	NM_004773	Zinc finger, HIT-type containing 3 (ZNHIT3)
2	XM_011516569	Zyxin (ZYX)
1		155 genes

### HE4 Induces DUSP6 Expression in PBMCs

**Figure 1:**
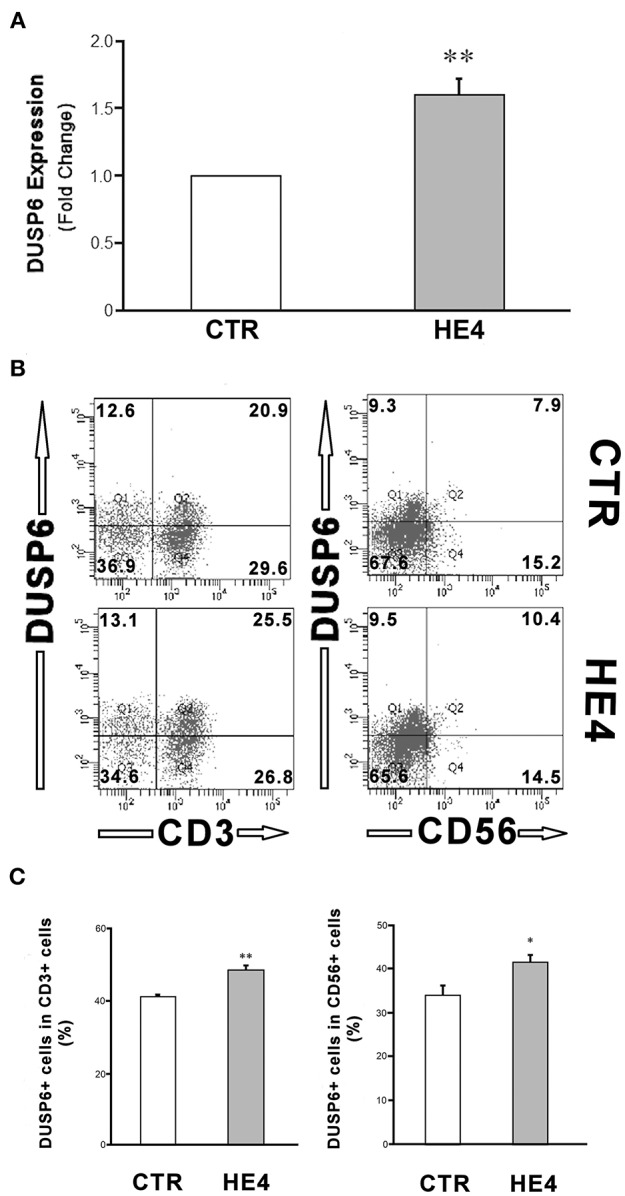
HE4 upregulates expression of DUSP6 in PBMCs. **(A)** DUSP6 transcription in response to a 6-hr incubation with 0.01 mg/mL rHE4 (HE4) or equivalent amount of PBS (CTR) were evaluated by triplicated trials of real time PCR using PBMCs from four individual donors. **(B)** Two-color flow cytometric analysis of PBMC following 24-h incubation with 0.01 mg/mL of rHE4 (HE4) or vehicle (CTR). 2D-scatterplots of DUSP6 (Alexa Fluor 647) and CD3 or CD56 (FITC) are shown. Numbers on the plots indicate mean percentages of the cell populations of the each tetrameric area from four independent experiments. **(C)** Bar graphs from flow cytometric analyses using PBMCs from four individual donors. The mean ± SEM are shown. *p < 0.05, **p < 0.01.

**Figure 2:**
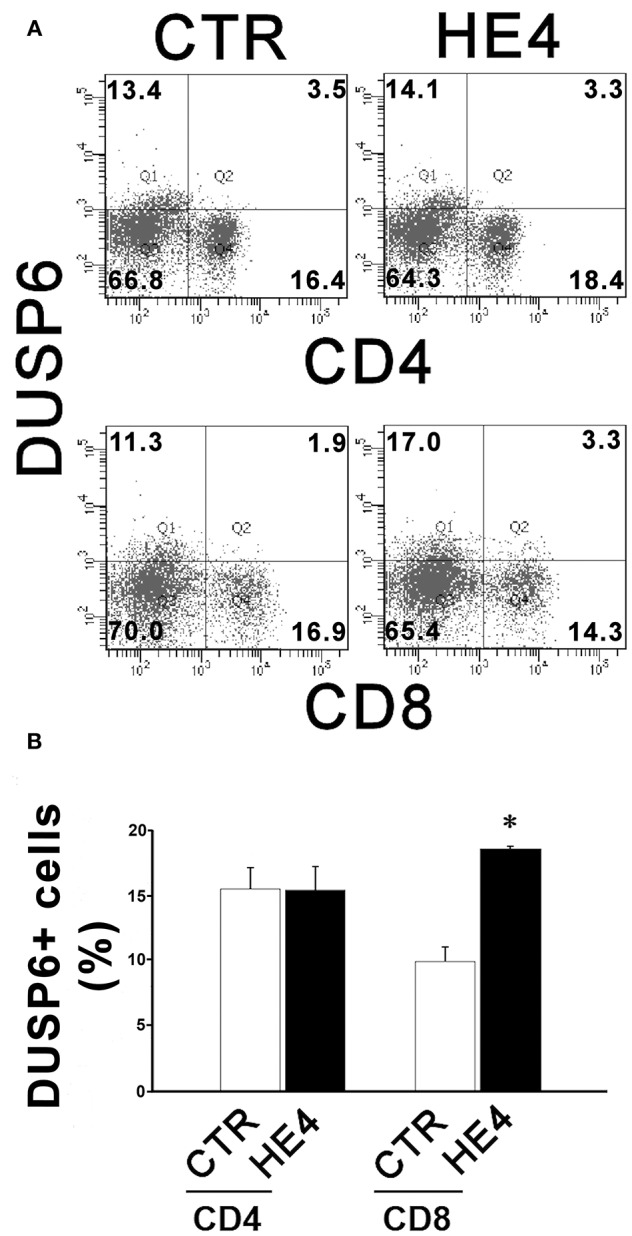
HE4 upregulates expression of DUSP6 in peripheral CD8^+^ T cells. Two-color flow cytometric analysis of PBMCs following 24-h incubation with 0.01 mg/mL of rHE4 (HE4) or vehicle (CTR). **(A)** 2D-scatterplots of DUSP6 (Alexa Fluor 647) and CD4 or CD8 (FITC) are shown. Numbers on the plots indicate mean percentages of the cell populations of the each tetrameric area from four independent experiments. **(B)** A bar graph from flow cytometric analyses using PBMCs from four individual donors. The mean using ± SEM are shown. *p < 0.01.

HE4-induced upregulation of DUSP6 in PBMCs was then confirmed via three modalities: quantitative PCR (qPCR), ELISA and flow cytometry. First, PBMCs were harvested after a 6 h exposure with recombinant human HE4 (rHE4; 0.01 μg/mL), revealing a 1.60 ± 0.13-fold increase (*p* < 0.01) in DUSP6 mRNA production (Figure 1A). The concentrations of DUSP6 in PBMC lysates (9.38 ± 0.62 vs. 15.62 ± 0.97 ng/mL, *p* < 0.01) and culture supernatants (0.77 ± 0.10 vs. 1.43 ± 0.14 ng/mL, *p* < 0.01) after a 24 h exposure to rHE4 were also increased (Table 2). PBMCs were then cultured with rHE4 for 24 h and collected for flow cytometry analysis. Protein expression of DUSP6 in CD3^+^ PBMCs (T cells) was found to be significantly increased with HE4 exposure (34.4 ± 0.6% vs. 47.0 ± 3.2% of total CD3^+^ cells; *p* < 0.05; Figures 1B,C left panel). DUSP6 expression in CD56^+^ cells (NK/T cells, NK cells) was also increased to a lesser extent (34.1 ± 2.3 vs. 41.7 ± 1.7% of total CD56^+^ cells; *p* < 0.05; Figures 1B,C right panel). Insignificant changes were observed in DUSP6 expression amongst CD14^+^ (monocytes) and CD19^+^ (B cells), as shown in Figure S4. In order to identify a T cell subset involved in the HE4 responsive induction of DUSP6, two-color flow cytometry using anti-DUSP6 antibody and anti-CD4 (helper T cell) or CD8 (cytotoxic T cell) antibodies were performed. As shown in Figures 2A,B, after a 24 h exposure to rHE4, CD8^+^ T cells (9.9 ± 0.8% vs. 1.9 ± 0.1%; *p* < 0.01) but not CD4^+^ T cells (15.6 ± 1.4% vs. 15.4 ± 1.5%) exhibited significant DUSP6 induction. These finding suggested that the CD8^+^ and CD56^+^ cytotoxic mononuclear cells were responsible for the HE4 responsive DUSP6 induction.

**Table 2:** DUSP6 concenrations in cell lysates and culture media of PBMC.

**Cell**	Lysates^*^	**Culture**	Media^**^
CTR	HE4	CTR	HE4
9.38 ± 0.62	15.62 ± 0.97^***^	0.77 ± 0.10	1.43 ± 0.14^***^
			ng/mL

* *In 2.5 mg/ml of total protein*.

** *In 5mL media of 5 × 10^6^ PBMC culture*.

*The mean ± SEM are shown*.

*** *p < 0.01 vs. CTR*.

### CD8^+^ and CD56^+^ Cytotoxic Lymphocytes Are Targets of HE4 Induced DUSP6

**Figure 3:**
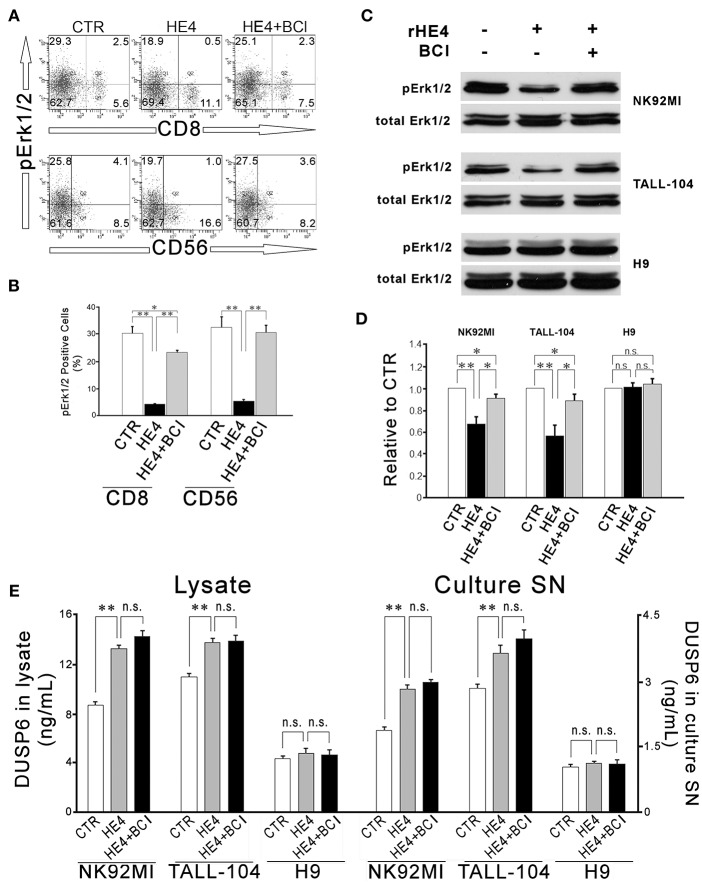
HE4 suppresses Erk1/2 phosphorylation in CD8^+^ and CD56^+^ cells via DUSP6 induction. Two-color flow cytometric analysis of PBMC following 24-hr incubation with PBS (CTR), rHE4 (0.01 mg /mL) and BCI (1 mM) as indicated. **(A)** 2-D scatterplots of phosphor-Erk1/2 (Alexa Fluor 647) and CD8 or CD56 (FITC) are shown. **(B)** Mean ± SEM from analyses of phosphor-Erk1/2 positive cells from four individual donors are shown in the bar graph. **(C)** Immunoblotting for phosphor-Erk1/2 in CD56^+^ NK92MI, CD8^+^ TALL-104 and CD4^+^ H9 cells following 1-h incubation with the conditioned media from 24-h PBMC culture with rHE4 (0.01 mg/mL) and BCI (1 mM) in the indicated combinations. Blots of total Erk1/2 are shown as loading controls. **(D)** Bar graph represents the relative band densities to controls. Mean ± SEM are shown (*n* = 4). **(E)** DUSP6 concentrations in cell lysates and culture supernatants (SN) after 24 h incubation with or without rHE4 (0.01 mg/mL). Mean ± SEM are shown (*n* = 4). *p < 0.05, **p < 0.01, n.s., not significant.

In order to identify effector cells for HE4 induction of DUSP6, two-color flow cytometry using antibodies against phosphor-Erk1/2 (pErk1/2) and CD4, CD8, CD14, CD19, and CD56 were performed. Significant decreases of pErk1/2^+^ populations were observed in CD8^+^ (30.2 ± 2.4% vs. 4.3 ± 0.2% in total CD8^+^ cells; *p* < 0.01) and CD56^+^ (32.3 ± 4.0% vs. 5.4 ± 0.6% in total CD56^+^ cells; *p* < 0.01) cells after a 24 h rHE4 (0.01 μg/mL) exposure. These decreases were abrogated by co-treatment with 1 μM of the DUSP6 inhibitor (E)-2-benzylidene-3-(cyclohexylamino)-2,3-dihydro-1H-inden-1-one (BCI) in both CD8^+^ cells and CD56^+^ cells (23.3 ± 0.7% and 30.5 ± 2.6%, respectively; Figures 3A,B). Insignificant changes were observed in pErk1/2^+^ populations in CD4^+^, CD14^+^, and CD19^+^ cells as shown in Figure S5.

Next, CD56^+^ NK cell line (NK92MI), CD8^+^ cytotoxic T cell line (TALL-104) and CD4^+^ helper T cell line (H9) were incubated with the conditioned media from a 24 h PBMC culture with or without rHE4 and BCI for 1 h. Residual rHE4 in the conditioned media was deprived by immunoprecipitation (Table S2). The lysates of the cells were used for western blotting to evaluate Erk1/2 phosphorylation. As shown in Figures 3C,D, 1 h incubation with the HE4 exposed PBMC conditioned media suppressed Erk1/2 phosphorylation in NK92MI cells (0.67 ± 0.07-fold vs. CTR, *p* < 0.01) and TALL-104 cells (0.56 ± 0.10-fold vs. CTR, *p* < 0.01) but not in H9 cells (1.01 ± 0.03-fold vs. CTR). The rHE4 responsive pErk1/2 suppressions were abrogated by the PBMC conditioned media from co-treatment with rHE4 and BCI in both NK92MI (0.90 ± 0.04-fold vs. CTR) and TALL-104 (0.89 ± 0.06-fold vs. CTR). Finally, the cell lines were seeded at 1 × 10^6^/mL density. Twelve hours later, the cells were deprived of serum overnight. The quiescent cells were incubated for 24 h with or without 0.01 μg/mL of rHE4, and then the concentrations of DUSP6 were determined in the cell lysates containing 2.5 mg/mL of proteins or culture supernatants (SN). As shown in Figure 3E, NK92MI and TALL-104 showed significant increases in DUSP6 concentrations in response to rHE4 exposure both in lysates and culture SN (KI, 92MI; lysate, 8.76 ± 0.27 vs. 13.33 ± 0.28 ng/mL, SN, 1.88 ± 0.08 vs. 2.83 ± 0.10, TALL-104; lysate, 11.00 ± 0.32 vs. 13.82 ± 0.33, SN, 2.85 ± 0.09 vs. 3.64 ± 0.19, *p* < 0.01). On the other hand, H9 showed no significant response to rHE4 (lysate, 4.44 ± 0.17 vs. 4.85 ± 0.38, SN, 1.04 ± 0.07 vs. 1.13 ± 0.05). These findings suggested that HE4 induced DUSP6 acts as an autocrine suppressor for Erk1/2 MAPK in CD8^+^ and CD56^+^ cytotoxic lymphocytes.

### HE4 Attenuates Ovarian Cancer Susceptibility to PBMC Mediated Cytotoxicity

In order to evaluate the impact of HE4 on PBMC cytotoxicity against cancer cells, the human ovarian tumor cell line, SKOV3, was co-cultured with PBMCs (5 × 10^6^/mL density). To minimize the effect of native HE4 produced by tumor cells, the SKOV3 cells were stably transfected with HE4 specific shRNA (shHE4). A clone of shRNA transfected cells used in this experiment was tested for its phenotype by western blotting (Figure S2). After incubation at 37°C for various lengths of time, concentrations of HE4 in the media were measured by ELISA (Table S3). HE4 concentrations in the media of the HE4 suppressed SKOV3 cells were below detection limit at any point up to 72 h (data not shown).

**Figure 4:**
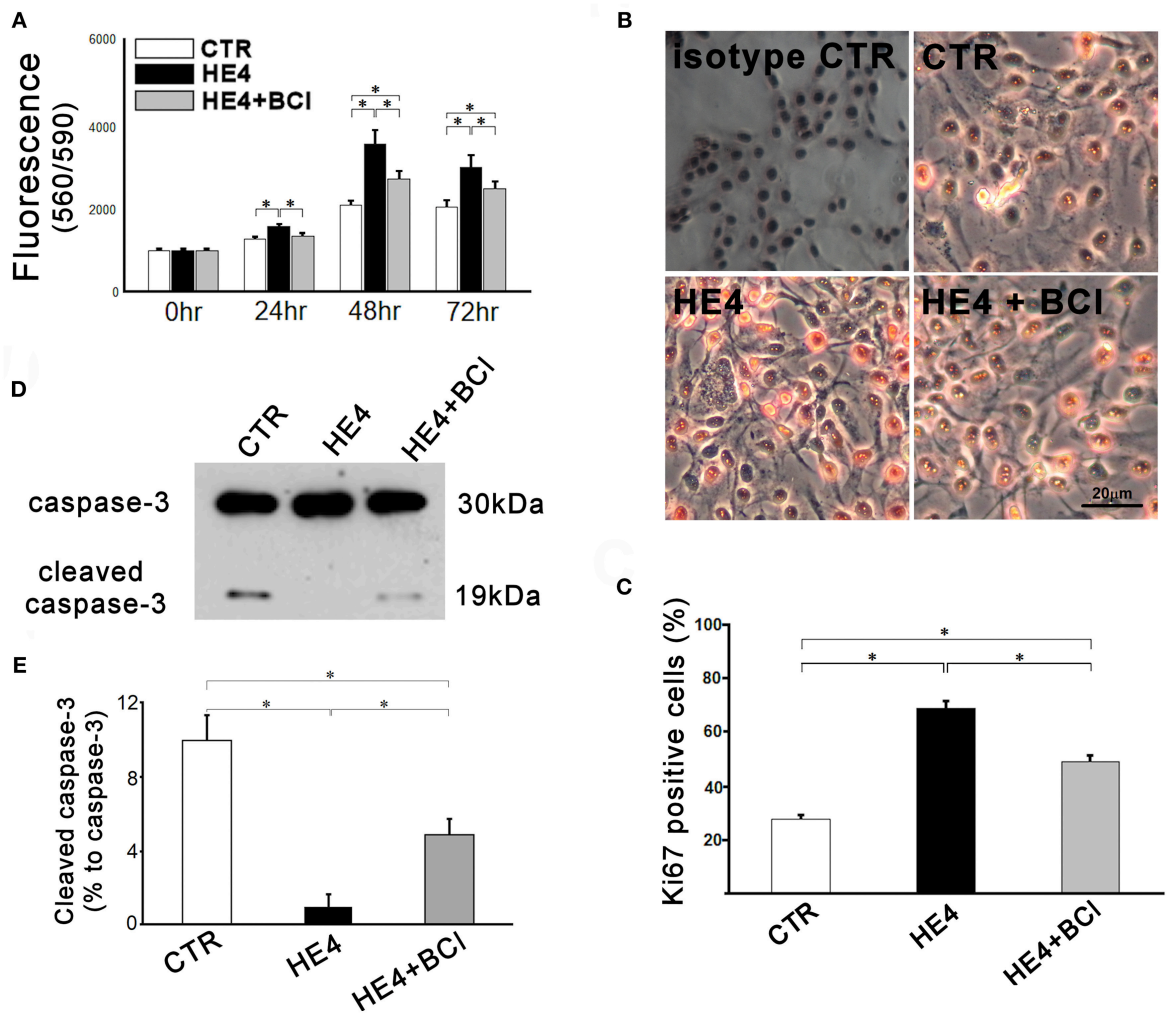
Responses of SKOV3 cells to co-culture with PBMCs were evaluated. Cells were co-cultured with PBMCs (5 × 10^6^/mL) alone (CTR) or PBMCs with rHE4 (0.01 mg /mL) and BCI (1 mM) in indicated combinations. **(A)** Cell proliferation was assessed at 24, 48, and 72 h of the culture (*n* = 10). **(B)** Ki67 immunohistochemistry staining was performed on SKOV3 cells co-cultured with PBMCs for 24 h. Ki67^+^ cells are identified with red nuclear staining. Cells incubated with normal mouse IgG1 were shown as an isotype control. **(C)** Bar graph represents the percentage of Ki67^+^ cells in total countable cells under 200x fields (*n* = 20). **(D)** A representative image of western blotting for intact and cleaved caspase-3 of SKOV3 lysates following 6-h PBMCs co-culture with of rHE4 (0.01 mg/mL) and BCI (1 mM) as indicated. **(E)** Bar graph represents the percentage of cleaved caspase-3 relative to intact caspase-3 (*n* = 4). Mean ± SEM are shown in the bar graphs. *p < 0.01.

After 12 h incubation, the effector cells (PBMCs) were washed away, and the target cells (SKOV3) were analyzed by three independent modalities: cell proliferation, Ki67 immunostaining, and western blotting for intact and cleaved caspase-3. First, SKOV3 cells co-cultured with PBMC suspensions containing 0.01 μg/mL of rHE4 showed significantly more proliferation than cells cultured with the rHE4 free suspensions at 24 (1222.70 ± 29.48 vs. 1517.98 ± 34.32, *p* < 0.01), 48 (2038.38 ± 55.94 vs. 3508.64 ± 164.98, *p* < 0.01) and 72 h (1983.33 ± 100.41 vs. 2935.89 ± 116.47, *p* < 0. 01), and the accelerated proliferations were partially abrogated by adding 1 μM of BCI to the culture (1295.68 ± 39.87, 2667.27 ± 95.13, and 2424.50 ± 105.70, at 24, 48, and 72 h, respectively Figure 4A).

Second, immunohistochemistry using anti-Ki67 was performed to evaluate the proliferation activities of SKOV3 cells in the presence of PBMCs with or without rHE4 and BCI for 24 h. Affinity purified normal mouse IgG1 (Santa Cruz Biotechnology, sc-3877), which was the identical isotype to the anti-Ki67 antibody, was used for isotype control. The number of Ki67 positive tumor cells in rHE4-containing PBMC suspension was higher than the cells in rHE4-free suspension, and the increased activity was partially attenuated by adding BCI to the culture (27.6 ± 1.7%, 68.5 ± 2.6%, and 48.9 ± 2.3%, respectively; Figures 4B,C). Incubation with BCI (1 μM) alone showed no significant effects on the proliferation of SKOV3 cells.

**Figure 5:**
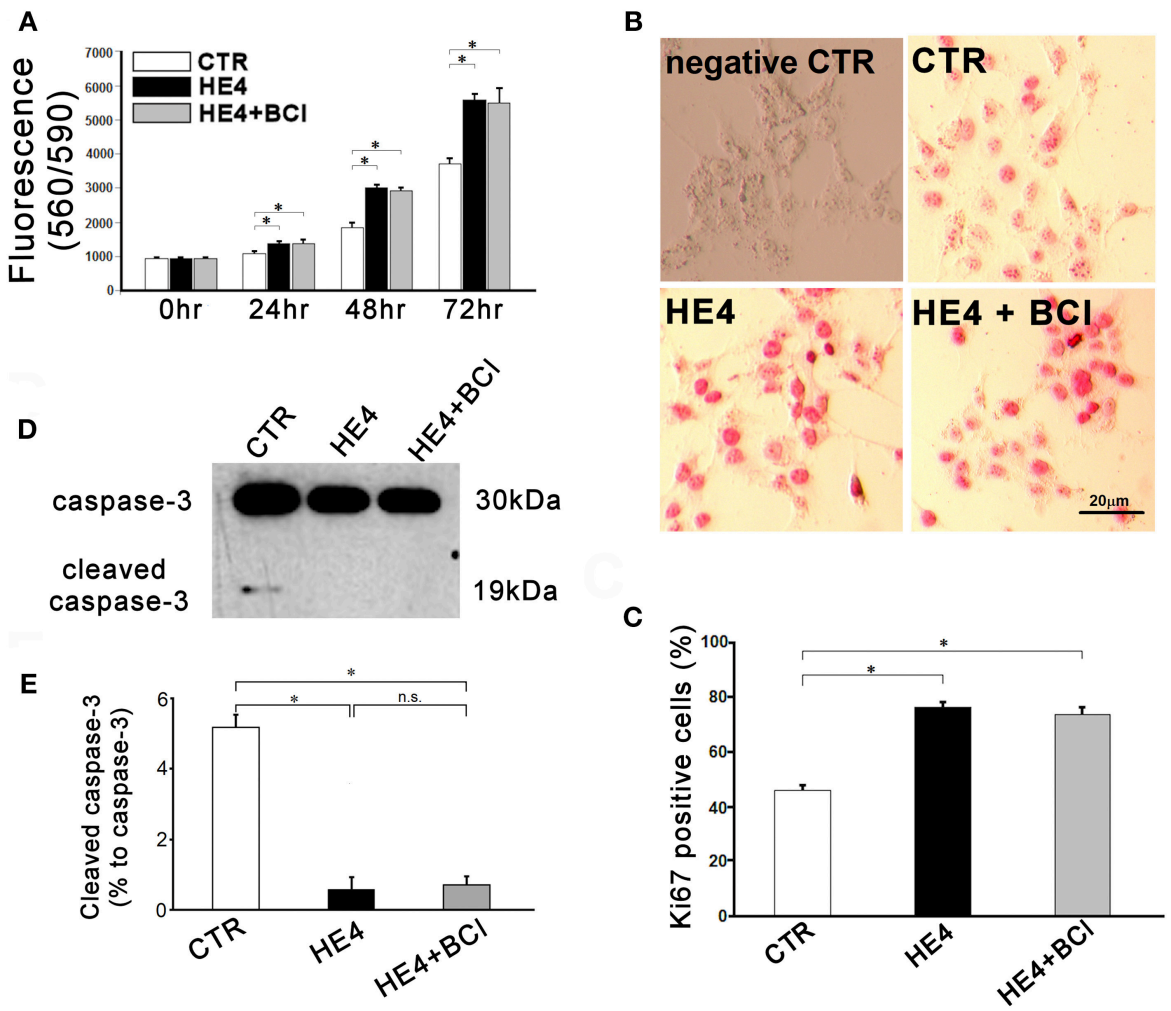
Responses of SKOV3 cells to co-culture with CD8^+^/CD56^+^ cell free PBMCs (5 × 10^6^ / mL) were evaluated. Cells were co-cultured with CD8^+^/CD56^+^ cell-free PBMCs alone or CD8^−^/CD56^−^ PBMCs with rHE4 (0.01 mg/mL) and BCI (1 mM) in indicated combinations. **(A)** Cell proliferation was assessed at 24, 48, and 72 h of the culture (*n* = 10). **(B)** Ki67 immunohistochemistry staining was performed on SKOV3 cells co-cultured with CD8^+^/CD56^+^ cell-free PBMCs for 24 hrs. Ki67^+^ cells are identified with red nuclear staining. Cells incubated with normal mouse IgG1 were shown as an isotype control. **(C)** Bar graph represents the percentage of Ki67^+^ cells in total countable cells under 200x fields (*n* = 20). **(D)** A representative image of western blotting for intact and cleaved caspase-3 of SKOV3 lysates following 6-h CD8^+^/CD56^+^ cell free PBMCs co-culture with of rHE4 (0.01 mg/mL) and BCI (1 mM) as indicated. **(E)** Bar graph represents the percentage of cleaved caspase-3 relative to intact caspase-3 (*n* = 4). Mean ± SEM are shown in the bar graphs. *p < 0.01, n.s., not significant.

Finally, protein lysates from the target cells were subjected to western blotting for caspase-3. As shown in Figures 4D,E, SKOV3/PBMC co-cultures with rHE4 led to a significant decrease in ratios of cleaved caspase-3 to intact caspase-3 (10.0 ± 1.4% vs. 1.0 ± 0.7%, *p* < 0.01), and the tolerance of target cells was partially reversed by adding BCI to the culture (4.9 ± 0.9%, *p* < 0.01 vs. CTR and HE4). Incubations with BCI (1 μM) alone showed no significant effects on SKOV3 cell proliferation, Ki67 expression, or apoptosis. These findings suggest that HE4 enhances tolerance of cancer cells against immunocompetent mononuclear cells via the up-regulation of DUSP6 in PBMCs. In order to confirm involvement of CD8^+^/CD56^+^ cytotoxic lymphocytes in the HE4 induced immunomodulation, the co-culture study was repeated using PBMCs deprived of CD8^+^/CD56^+^ cells (Figure S3). As shown in Figures 5A–E, all the effects of BCI shown in Figure 4 were erased in the CD8^+^/CD56^+^ cell free co-cultures, suggesting that cytotoxic lymphocytes play a pivotal role in immunoediting by DUSP6 up-regulation in response to HE4 exposure.

## Discussion

Several studies from our laboratory and elsewhere have revealed multidimensional roles for HE4 in the pathogenesis of ovarian cancer, including the promotion of tumor growth, chemoresistance, anti-estrogen resistance, invasion, migration, and adhesion (Lu et al., 2012; Zhuang et al., 2013, 2014; Zhu et al., 2013, 2016; Lokich et al., 2014; Moore et al., 2014; Wang et al., 2015; Ribeiro et al., 2016; Lee et al., 2017). In this present study, we have begun to delineate another vital function of HE4 in disrupting immune cell function, which has implications for immune system targeting of tumor cells. DUSP6, which we found to be upregulated by rHE4 treatment in CD8^+^ T cells and CD56^+^ NK cell subsets of PBMCs, is likely one key mediator of this effect in these immune cell subsets.

DUSP6 is a member of the DUSP family that dephosphorylates threonine and tyrosine residues on MAPK substrates. It specifically dephosphorylates ERK, a member of the MAPK family that also includes p38 and JNK. MAPKs are activated by growth factors, cytokines, integrin ligands, and stress signals to regulate growth, survival, apoptosis, and immune response in diverse cell types. Interestingly, DUSP6 is expressed at low levels in resting cells and is actually stimulated by ERK activation, promoting a negative feedback loop on ERK activity (Bermudez et al., 2010). This early response of DUSP6 to ERK activation could explain the apparently contradictory activation of ERK by HE4 in cancer cells (Lu et al., 2012; Zhu et al., 2013; Ribeiro et al., 2016; Lee et al., 2017) and our current results here showing HE4 upregulation of DUSP6 expression leading to suppression of ERK phosphorylation in PBMC subsets.

Several reports reveal a role for DUSP6 in development, organogenesis, and cancer (Bermudez et al., 2010). However, its effect on cancer progression is highly dependent upon the type of cancer and even the stage. For example, in pancreatic cancer, it is upregulated in early stages but is often completely diminished as the tumor progresses toward the invasive ductal carcinoma state (Furukawa et al., 2005). In lung cancer, it has been shown to act as a tumor suppressor (Okudela et al., 2009); conversely, it is upregulated in glioblastoma and HER2-positive breast cancer (Lucci et al., 2010; Messina et al., 2011). One report found that its downregulation in ovarian cancer results in hyperactivation of ERK and subsequent chemoresistance (Chan et al., 2008). These discrepancies are likely due to variable deregulation of ERK signaling and compensatory pathways that are highly context dependent (Bermudez et al., 2010).

While much is still not understood regarding the role of tumor-produced DUSP6 on tumorigenesis, the function of DUSP6 originating from immune cells is even less well studied. Other members of the DUSP family, including DUSP1, DUSP2, and DUSP10, are known to have roles in immune response (Bermudez et al., 2010), and a few reports suggest that DUSP6 does as well. Elevated DUSP6 was shown to cause downregulation of ERK phosphorylation in CD4^+^ T cells in elderly individuals, who have suppressed immune responses (Li et al., 2012). Another report confirmed this age associated rise in CD4^+^ T cell DUSP6 expression, and found that young immunosuppressed patients with end stage renal disease have DUSP6 levels comparable to elderly healthy individuals (Huang et al., 2017). One study found that DUSP6 downregulates ERK activity in CD4^+^ T cells and increases their regulatory T cell functions (Bertin et al., 2015). Together, these reports suggest that higher levels of DUSP6 contribute to immune suppression. It has also been shown that DUSP6 is downregulated in T cells upon IL-2 withdrawal (Chechlinska et al., 2009), and IL-2 was found to upregulate DUSP6 gene expression in T cells (Kovanen et al., 2005). Since IL-2 stimulates cytotoxic T cell expansion and activation as well as that of immune suppressive regulatory T cells (Boyman and Sprent, 2012), it remains to be determined how the IL-2 responsiveness of DUSP6 plays into its apparent effect on immune suppression, and how this relates to tumor immune response.

Although much remains unknown regarding the specific effects of DUSP6 on cancer progression and tumor immunity, our findings begin to reveal some novel insights. We report for the first time that HE4-mediated upregulation of DUSP6 in CD8^+^ T cell and CD56^+^ NK cell subsets of PBMC cells leads to the inhibition of their cytotoxic activity against SKOV3 ovarian cancer cells. While DUSP6 has been connected to immune function of CD4^+^ T cells, our results reveal that the subsets of lymphocytes affected by DUSP6 are context dependent. Further investigation into the inhibitory effects of DUSP6 in these different populations will be illuminating. Moreover, we have begun to establish HE4 as a critical regulator of immune cell function, which deepens our understanding of the mechanistic role HE4 plays in ovarian cancer pathogenesis.

## Data Availability

All datasets generated for this study are included in the manuscript and/or the supplementary files.

## Ethics Statement

This study was carried out in accordance with The National Statement on Ethical Conduct in Human Research with written informed consent from a donor of the blood sample. The study was reviewed and approved by Institutional Review Board (IRB) of Women and Infants Hospital.

## Author Contributions

NJ, JR, and NY designed experiments and executed all experiments with assistance from MO and EC. NY developed all the stable clones. NJ, JR, and NY prepared this manuscript. RR-T, K-KK, CC, PD, RM, RS, and TZ all contributed conceptually this manuscript. All authors reviewed and approved the manuscript.

### Conflict of Interest Statement

The authors declare that the research was conducted in the absence of any commercial or financial relationships that could be construed as a potential conflict of interest.

## Footnotes

^1^*What Are the Key Statistics About Ovarian Cancer?* Available online at: https://www.cancer.org/cancer/ovarian-cancer/about/key-statistics.html

^2^*SEER Cancer Statistics Review, 1975-2014, National Cancer Institute*. Available online at: https://seer.cancer.gov/csr/1975_2014/

## References

[B1] Angioli R. Capriglione S. Aloisi A. Guzzo F. Luvero D. Miranda A.. (2014). Can HE4 predict platinum response during first-line chemotherapy in ovarian cancer? Tumor Biol. 35, 7009–7015. 10.1007/s13277-014-1836-x 24748235

[B2] Bermudez O. Pagès G. Gimond C. (2010). The dual-specificity MAP kinase phosphatases: critical roles in development and cancer. Am. J. Physiol. Cell Physiol. 299, C189–C202. 10.1152/ajpcell.00347.2009 20463170

[B3] Bertin S. Lozano-Ruiz B. Bachiller V. García-Martínez I. Herdman S. Zapater P.. (2015). Dual-specificity phosphatase 6 regulates CD4^+^ T-cell functions and restrains spontaneous colitis in IL-10-deficient mice. Mucosal. Immunol. 8, 505–515. 10.1038/mi.2014.84 25227984 PMC4363301

[B4] Bingle L. Singleton V. Bingle C. D. (2002). The putative ovarian tumour marker gene HE4 (WFDC2), is expressed in normal tissues and undergoes complex alternative splicing to yield multiple protein isoforms. Oncogene 21, 2768–2773. 10.1038/sj.onc.1205363 11965550

[B5] Boyman O. Sprent J. (2012). The role of interleukin-2 during homeostasis and activation of the immune system. Nat. Rev. Immunol. 12, 180–190. 10.1038/nri3156 22343569

[B6] Chan D. W. Liu V. W. Tsao G. S. Yao K. M. Furukawa T. Chan K. K.. (2008). Loss of MKP3 mediated by oxidative stress enhances tumorigenicity and chemoresistance of ovarian cancer cells. Carcinogenesis 29, 1742–1750. 10.1093/carcin/bgn167 18632752

[B7] Chechlinska M. Siwicki J. K. Gos M. Oczko-Wojciechowska M. Jarzab M. Pfeifer A.. (2009). Molecular signature of cell cycle exit induced in human T lymphoblasts by IL-2 withdrawal. BMC Genomics 10:261. 10.1186/1471-2164-10-261 19505301 PMC2706892

[B8] Chudecka-Głaz A. M. Cymbaluk-Płoska A. A. Menkiszak J. L. Sompolska-Rzechuła A. M. Tołoczko-Grabarek A. I. Rzepka-Górska I. A. (2014). Serum HE4, CA125, YKL-40, bcl-2, cathepsin-L and prediction optimal debulking surgery, response to chemotherapy in ovarian cancer. J. Ovarian Res. 7:62. 10.1186/1757-2215-7-62 25018782 PMC4094548

[B9] Curran M. A. Montalvo W. Yagita H. Allison J. P. (2010). PD-1 and CTLA-4 combination blockade expands infiltrating T cells and reduces regulatory T and myeloid cells within B16 melanoma tumors. Proc. Natl. Acad. Sci. U.S.A. 107, 4275–4280. 10.1073/pnas.0915174107 20160101 PMC2840093

[B10] Furukawa T. Fujisaki R. Yoshida Y. Kanai N. Sunamura M. Abe T.. (2005). Distinct progression pathways involving the dysfunction of DUSP6/MKP-3 in pancreatic intraepithelial neoplasia and intraductal papillary-mucinous neoplasms of the pancreas. Mod. Pathol. 18, 1034–1042. 10.1038/modpathol.3800383 15832194

[B11] Hellström I. Raycraft J. Hayden-Ledbetter M. Ledbetter J. A. Schummer M. McIntosh M.. (2003). The HE4 (WFDC2) protein is a biomarker for ovarian carcinoma. Cancer Res. 63, 3695–3700. 12839961

[B12] Holmgaard R. B. Zamarin D. Munn D. H. Wolchok J. D. Allison J. P. (2013). Indoleamine 2,3-dioxygenase is a critical resistance mechanism in antitumor T cell immunotherapy targeting CTLA-4. J. Exp. Med. 210, 1389–1402. 10.1084/jem.20130066 23752227 PMC3698523

[B13] Huang L. Litjens N. H. R. Kannegieter N. M. Klepper M. Baan C. C. Betjes M. G. H. (2017). pERK-dependent defective TCR-mediated activation of CD4+ T cells in end-stage renal disease patients. Immun. Ageing. 14:14. 10.1186/s12979-017-0096-1 28642802 PMC5477144

[B14] Kim A. Ueda Y. Naka T. Enomoto T. (2012). Therapeutic strategies in epithelial ovarian cancer. J. Exp. Clin. Cancer Res. 31:14. 10.1186/1756-9966-31-14 22330607 PMC3309949

[B15] Kovanen P. E. Young L. Al-Shami A. Rovella V. Pise-Masison C. A. Radonovich M. F.. (2005). Global analysis of IL-2 target genes: identification of chromosomal clusters of expressed genes. Int. Immunol. 17, 1009–1021. 10.1093/intimm/dxh283 15980098

[B16] Lee S. Choi S. Lee Y. Chung D. Hong S. Park N. (2017). Role of human epididymis protein 4 in chemoresistance and prognosis of epithelial ovarian cancer. J. Obs. Gynaecol. Res. 43, 220–227. 10.1111/jog.13181 27862665

[B17] Li G. Yu M. Lee W. W. Tsang M. Krishnan E. Weyand C. M.. (2012). Decline in miR-181a expression with age impairs T cell receptor sensitivity by increasing DUSP6 activity. Nat. Med. 18, 1518–1524. 10.1038/nm.2963 23023500 PMC3466346

[B18] Lokich E. Singh R. K. Han A. Romano N. Yano N. Kim K.. (2014). HE4 expression is associated with hormonal elements and mediated by importin-dependent nuclear translocation. Sci. Rep. 4:5500. 10.1038/srep05500 24975515 PMC4074789

[B19] Lu R. Sun X. Xiao R. Zhou L. Gao X. Guo L. (2012). Human epididymis protein 4 (HE4) plays a key role in ovarian cancer cell adhesion and motility. Biochem. Biophys. Res. Commun. 419, 274–280. 10.1016/j.bbrc.2012.02.008 22342977

[B20] Lucci M. A. Orlandi R. Triulzi T. Tagliabue E. Balsari A. Villa-Moruzzi E. (2010). Expression profile of tyrosine phosphatases in HER2 breast cancer cells and tumors. Cell Oncol. 32, 61–372. 10.3233/CLO-2010-0520 20413845 PMC4619248

[B21] Messina S. Frati L. Leonetti C. Zuchegna C. Di Zazzo E. Calogero A.. (2011). Dual-specificity phosphatase DUSP6 has tumor-promoting properties in human glioblastomas. Oncogene 30, 3813–3820. 10.1038/onc.2011.99 21499306

[B22] Mittica G. Genta S. Aglietta M. Valabrega G. (2016). Immune checkpoint inhibitors: a new opportunity in the treatment of ovarian cancer? Int. J. Mol. Sci. 17:E1169. 10.3390/ijms17071169 27447625 PMC4964540

[B23] Moore R. G. Hill E. K. Horan T. Yano N. Kim K. MacLaughlan S.. (2014). HE4 (WFDC2) gene overexpression promotes ovarian tumor growth. Sci. Rep. 4:3574. 10.1038/srep03574. 24389815 PMC3880958

[B24] Moore R. G. McMeekin D. S. Brown A. K. DiSilvestro P. Miller M. C. Allard W. J.. (2009). A novel multiple marker bioassay utilizing HE4 and CA125 for the prediction of ovarian cancer in patients with a pelvic mass. Gynecol. Oncol. 112, 40–46. 10.1016/j.ygyno.2008.08.031 18851871 PMC3594094

[B25] Moore R. G. Miller M. C. Steinhoff M. M. Skates S. J. Lu K. H. Lambert-Messerlian G.. (2012). Serum HE4 levels are less frequently elevated than CA125 in women with benign gynecologic disorders. Am. J. Obstet. Gynecol. 206, 351.e1–8. 10.1016/j.ajog.2011.12.029 22284961 PMC3985608

[B26] Okudela K. Yazawa T. Woo T. Sakaeda M. Ishii J. Mitsui H.. (2009). Down-regulation of DUSP6 expression in lung cancer: its mechanism and potential role in carcinogenesis. Am. J. Pathol. 175, 867–881. 10.2353/ajpath.2009.080489 19608870 PMC2716981

[B27] Ribeiro J. R. Schorl C. Yano N. Romano N. Kim K. K. Singh R. K.. (2016). HE4 promotes collateral resistance to cisplatin and paclitaxel in ovarian cancer cells. J. Ovarian Res. 9:28. 10.1186/s13048-016-0240-0 27184254 PMC4869286

[B28] Song J. X. Cao W. L. Li F. Q. Shi L. N. Jia X. (2012). Anti-Sp17 monoclonal antibody with antibody-dependent cell-mediated cytotoxicity and complement-dependent cytotoxicity activities against human ovarian cancer cells. Med. Oncol. 29, 2923–2931. 10.1007/s12032-011-0137-0 22198696

[B29] Vallius T. Hynninen J. Auranen A. Carpén O. Matomäki J. Oksa S.. (2014). Serum HE4 and CA125 as predictors of response and outcome during neoadjuvant chemotherapy of advanced high-grade serous ovarian cancer. Tum. Biol. 35, 12389–12395. 10.1007/s13277-014-2553-1 25190018

[B30] Volpe V. O. Klufas D. M. Hegde U. Grant-Kels J. M. (2017). The new paradigm of systemic therapies for metastatic melanoma. J. Am. Acad. Dermatol. 77, 356–368. 10.1016/j.jaad.2017.04.1126 28711086

[B31] Wang H. Zhu L. Gao J. Hu Z. Lin B. (2015). Promotive role of recombinant HE4 protein in proliferation and carboplatin resistance in ovarian cancer cells. Oncol. Rep. 33, 403–412. 10.3892/or.2014.3549 25354091

[B32] Yap T. A. Carden C. P. Kaye S. B. (2009). Beyond chemotherapy: targeted therapies in ovarian cancer. Nat. Rev. Cancer 9, 167–181. 10.1038/nrc2583 19238149

[B33] Zhao X. Subramanian S. (2018). Oncogenic pathways that affect antitumor immune response and immune checkpoint blockade therapy. Pharmacol. Ther. 181, 76–84. 10.1016/j.pharmthera.2017.07.004 28720430

[B34] Zhu L. Zhuang H. Wang H. Tan M. Schwab C. L. Deng L.. (2016). Overexpression of HE4 (human epididymis protein 4) enhances proliferation, invasion and metastasis of ovarian cancer. Oncotarget 7, 729–744. 10.18632/oncotarget.6327 26575020 PMC4808029

[B35] Zhu Y. F. Gao G. L. Tang S. B. Zhang Z. D. Huang Q. S. (2013). Effect of WFDC 2 silencing on the proliferation, motility and invasion of human serous ovarian cancer cells *in vitro*. Asian Pac. J. Trop. Med. 6, 265–272. 10.1016/S1995-7645(13)60055-3 23608327

[B36] Zhuang H. Gao J. Hu Z. Liu J. Liu D. Lin B. (2013). Co-expression of Lewis y antigen with human epididymis protein 4 in ovarian epithelial carcinoma. PLoS ONE 8:e68994. 10.1371/journal.pone.0068994 23894390 PMC3718801

[B37] Zhuang H. Hu Z. Tan M. Zhu L. Liu J. Liu D. (2014). Overexpression of Lewis y antigen promotes human epididymis protein4-mediated invasion and metastasis of ovarian cancer cells. Biochimie 105, 91–98. 10.1016/j.biochi.2014.06.022 24998328

