# Supplementary material for: Human Epididymis Secretory Protein 4 (HE4) Compromises Cytotoxic Mononuclear Cells via Inducing Dual Specificity Phosphatase 6
Source: Front Pharmacol. 2019 Mar 19;10:216. doi: 10.3389/fphar.2019.00216 (PMC6433991; doi:10.3389/fphar.2019.00216)
Supplement: Supplementary file 1 [file Table_1.docx]

**HE4 compromises cytotoxic mononuclear cells via inducing** **dual specificity phosphatase 6 secretion**

Nicole E. James, Matthew T. Oliver, Jennifer R. Ribeiro, Evelyn Cantillo, Rachael B. Rowswell-Turner, Kyu-Kwang Kim, Clinton O. Chichester III, Paul A. DiSilvestro, Richard G. Moore, Rakesh K. Singh, Naohiro Yano and Ting, C. Zhao

**Supplemental data; list of the material included**

1. Table S1
2. Table S2
3. Table S3
4. Figure S1A-C
5. Figure S2
6. Figure S3
7. Figure S4
8. Figure S5

| **Table S1** Summary of qPCR primer sequences | |  |
| --- | --- | --- |
| **genes** | **forward primer** | **reverse primer** |
| M13 | CGCCAGGGTTTTCCCAGTCACGAC | AGCGGATAACAATTTCACACAGGA |
| DUSP6 | CTCAAGAAGCTCAAGGACGAG | TAGGCATCGTTCATCGACAG |
| GAPDH | GACAACTTTGGTATCGTGGAAGG | AGGCAGGGATGATGTTCTGG |

1. A diagram of subtractive hybridization of differential PCR products


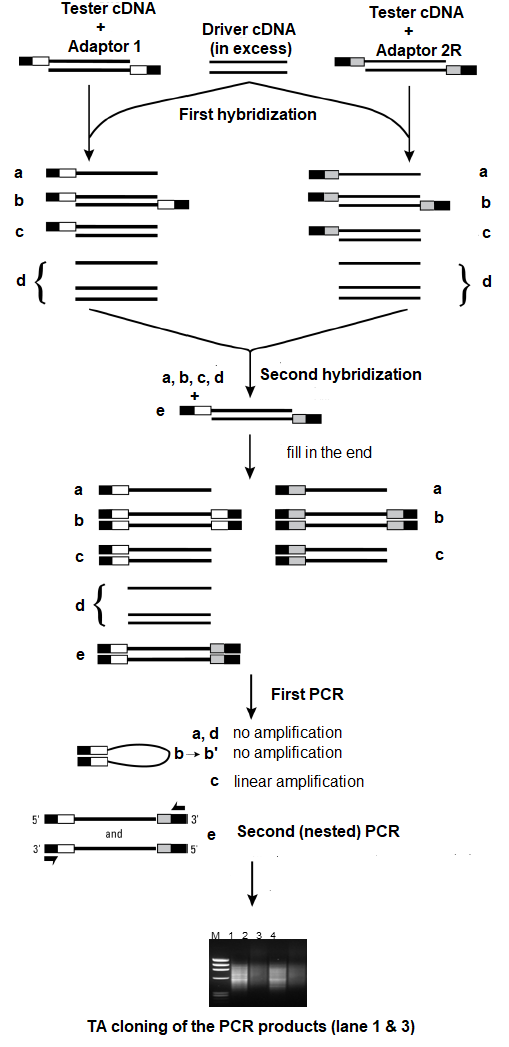
The tester and driver cDNAs are synthesized from poly A^+^ RNA generated from control and HE4 treated PBMCs. The tester and driver cDNAs are each digested with a restriction enzyme, Rsa I to yield shorter, blunt-ended molecules. The tester cDNA is then subdivided into two portions, and each is ligated with a different cDNA adaptor. The ends of the adaptor do not contain a phosphate group, so only one strand of each adaptor attaches to the 5' ends of the cDNA. The two adaptors have stretches of identical sequence to allow annealing of the PCR primer once the recessed ends have been filled in.

**First hybridization**

An excess of driver is added to each sample of tester. The samples are then heat denatured and allowed to anneal, generating the type **a**, **b**, **c**, and **d** molecules in each sample. The concentration of high- and low-abundance sequences is equalized among the type **a** molecule because reannealing is faster for the more abundant molecules due to the second-order kinetics of hybridization. At the same time, type **a** molecule are significantly enriched for differentially expressed sequences while cDNAs that are not differentially expressed form type **c** molecules with the driver.

**Second hybridization**

The two primary hybridization samples are mixed together without denaturing in order to generate PCR templates from differentially expressed sequences. Only the remaining equalized and subtracted single strand tester cDNAs can re-associate and form new type **e** hybrids. These new hybrids are double stranded tester molecules with different ends, which correspond to the sequences of Adaptors 1 and 2R. Then, fresh denatured driver cDNA is added (again, without denaturing the subtraction mix) to further enrich fraction **e** for differentially expressed sequences. After filling in the ends by DNA polymerase, the type e molecules—the differentially expressed tester sequences—have different annealing sites for the nested primers on their 5' and 3' ends.

**First PCR amplification**

The entire population of molecules is then subjected to suppression PCR, which exponentially amplifies only the desired differentially expressed sequences. During this PCR, type **a** and **d** molecules are missing primer annealing sites, and thus cannot be amplified. Due to the suppression PCR effect, most type **b** molecules form a pan-like structure that prevents their exponential amplification. Type **c** molecules have only one primer annealing site and amplify linearly, and only type **e** molecules—the equalized, differentially expressed sequences with two different adaptors—amplify exponentially.

**Second PCR amplification**

A secondary PCR amplification is performed using nested primers to further reduce any background PCR products and enrich for differentially expressed sequences.

1. TA cloning of the second PCR products

The PCR products were cloned into TA vector and subject to PCR direct sequencing

**
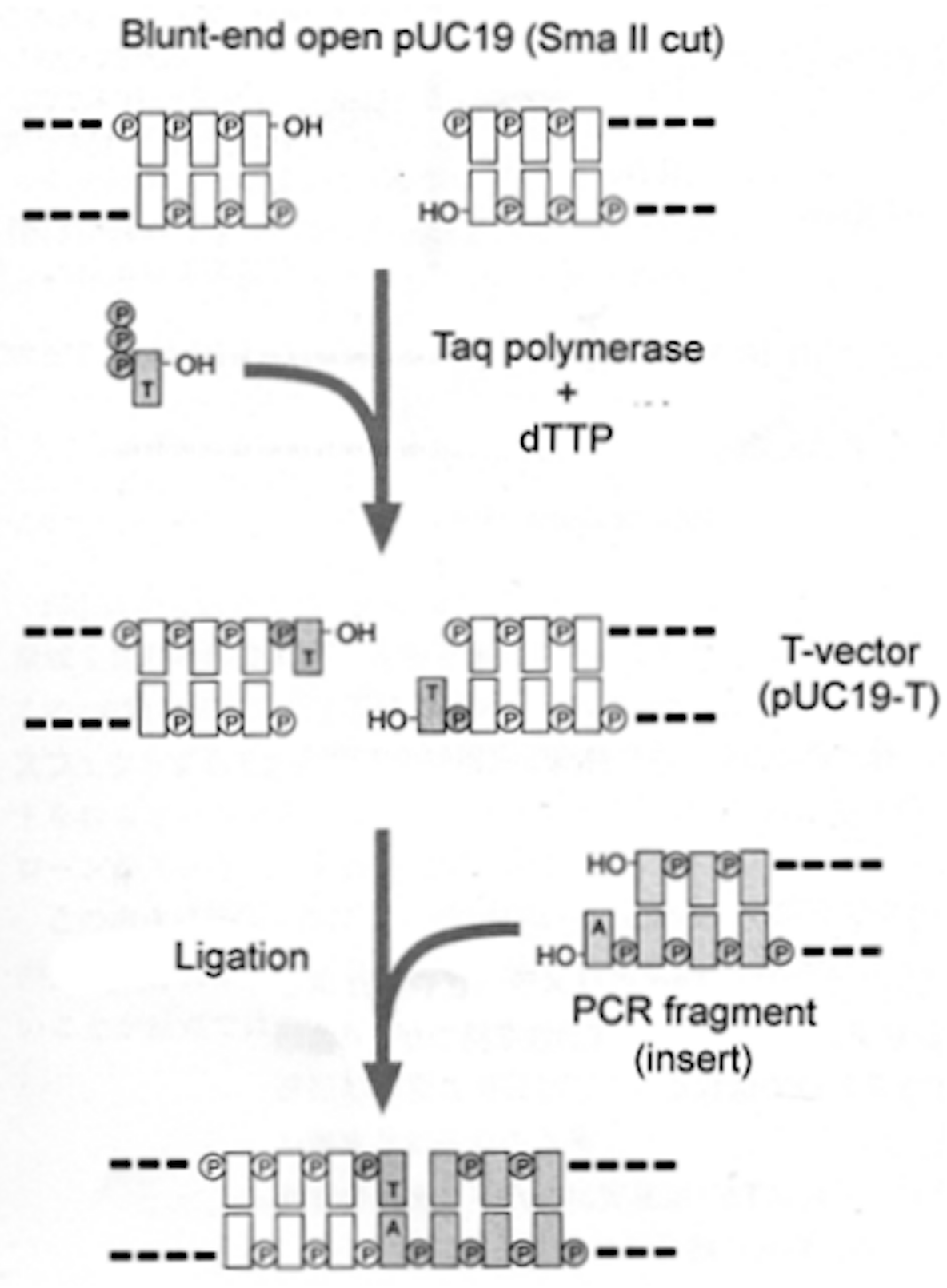
**

1.
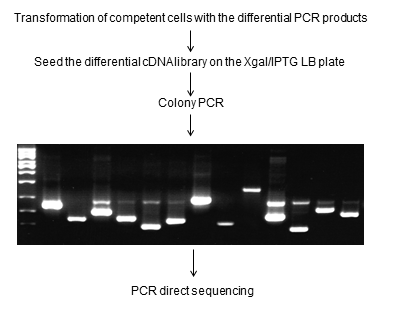
A flowchart of cloning to construct a library

**bp**

**1000**

**300**

**500**

**700**

Figure S2

**
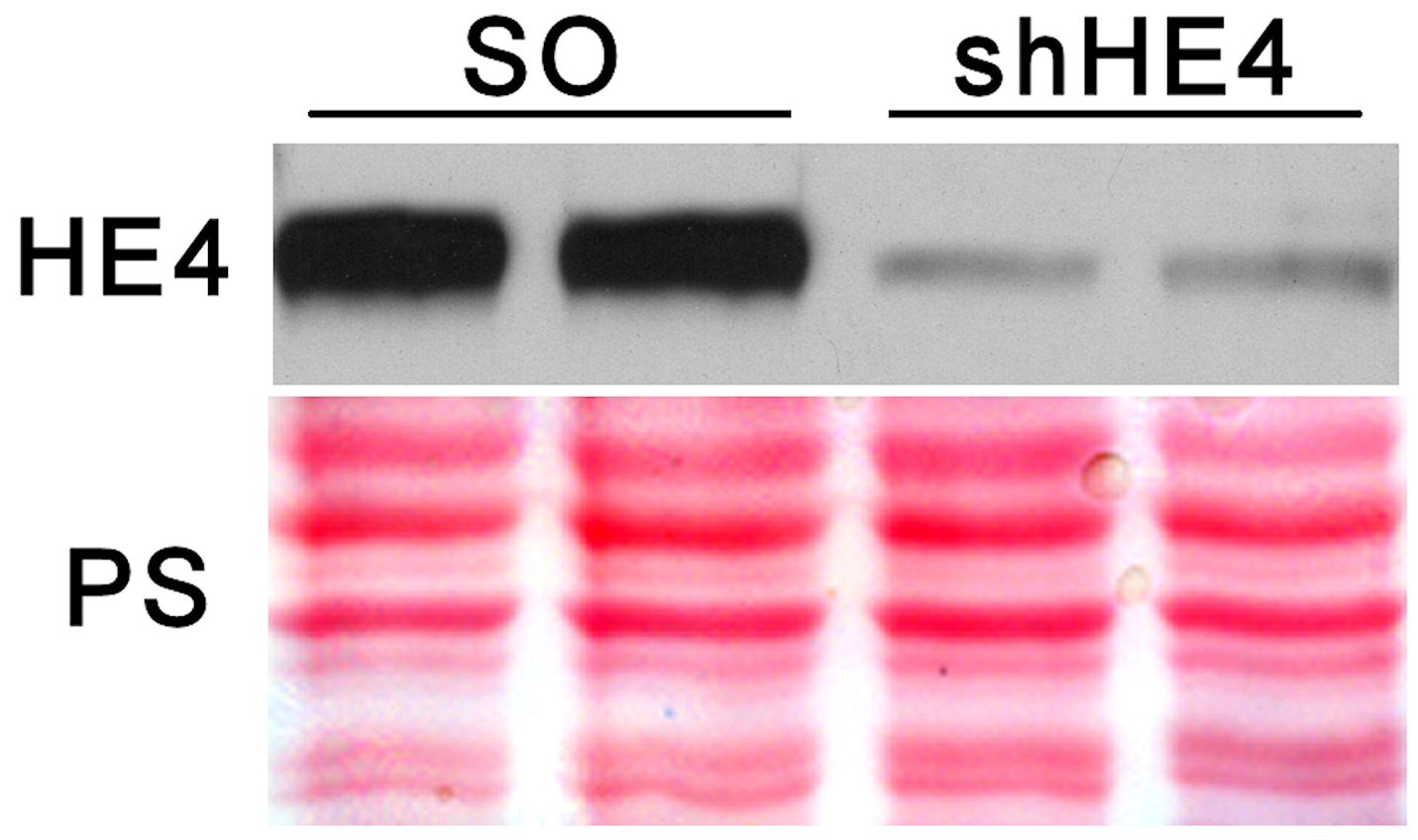
**

Western blotting of lysates from SKOV3 cells transfected with shRNA against HE4 (shHE4) or scrambled oligo (SO). Ponceau S stained membrane is shown as a loading control.

Figure S3

Two-color flow cytometric analysis of PBMCs before and after CD8 and CD56 positive cell depletion. Numbers on the plots indicate mean percentages of the cell populations of each tetrameric area from four independent experiments. (A) 2D-scatterplots of CD56 (PE) and CD8 (FITC) are shown. (B) The mean ± SEM are shown in the bar graph (n = 4).

Figure S4

**A B**
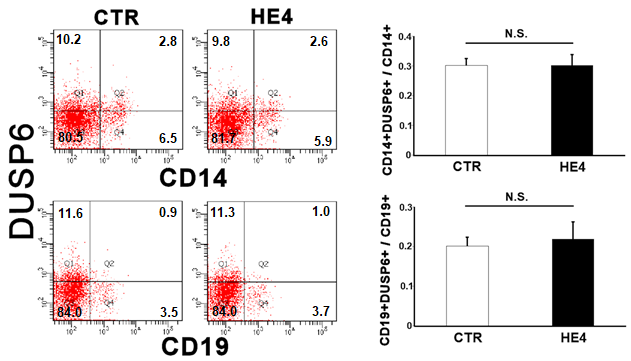


Two-color flow cytometric analysis of PBMCs after 24-h incubation with 0.01 μg/mL rHE4 (HE4) or vehicle (CTR). (A) 2D-scatterplots and graphs of DUSP6 (Alexa Fluor 647) and CD14 or CD19 (FITC) staining are shown. Numbers on the plots indicate mean percentages of the cell populations of each tetrameric area from four independent experiments. (B) The mean ± SEM are shown in the bar graph (n = 4). No significant differences in DUSP6 expression were observed in CD14^+^ cells (monocytes) and CD19^+^ cells (B cells).

Figure S5


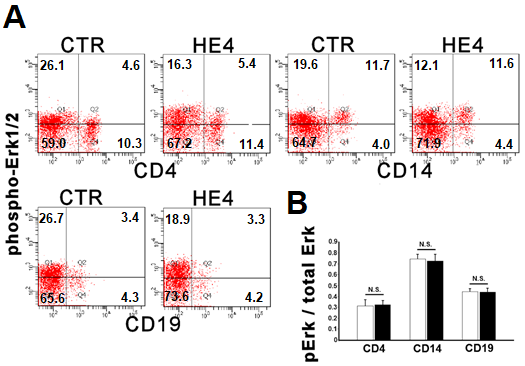


Two-color flow cytometric analysis of PBMCs after 24-hr incubation with 0.01 μg/mL rHE4 (HE4) or vehicle (CTR). (A) 2D-scatterplots and graphs of phosphor-Erk1/2 (Alexa Fluor 647) and CD4, CD14 or CD19 (FITC) staining are shown. (B) The mean ± SEM are shown in the bar graph (n = 4). No significant differences in Erk1/2 phosphorylation were observed in CD4^+^ cells (helper T cells), CD14^+^ cells (monocytes) and CD19^+^ cells (B cells).
